# Design Principles for the Acceptor Units in Donor–Acceptor Conjugated Polymers

**DOI:** 10.1021/acsomega.2c04713

**Published:** 2022-10-18

**Authors:** Tuǧba Hacıefendioǧlu, Erol Yildirim

**Affiliations:** † Department of Chemistry, Middle East Technical University, 06800 Ankara, Turkey; ‡ Department of Polymer Science and Technology, Middle East Technical University, 06800 Ankara, Turkey; § Department of Micro- and Nanotechnology, Middle East Technical University, 06800 Ankara, Turkey

## Abstract

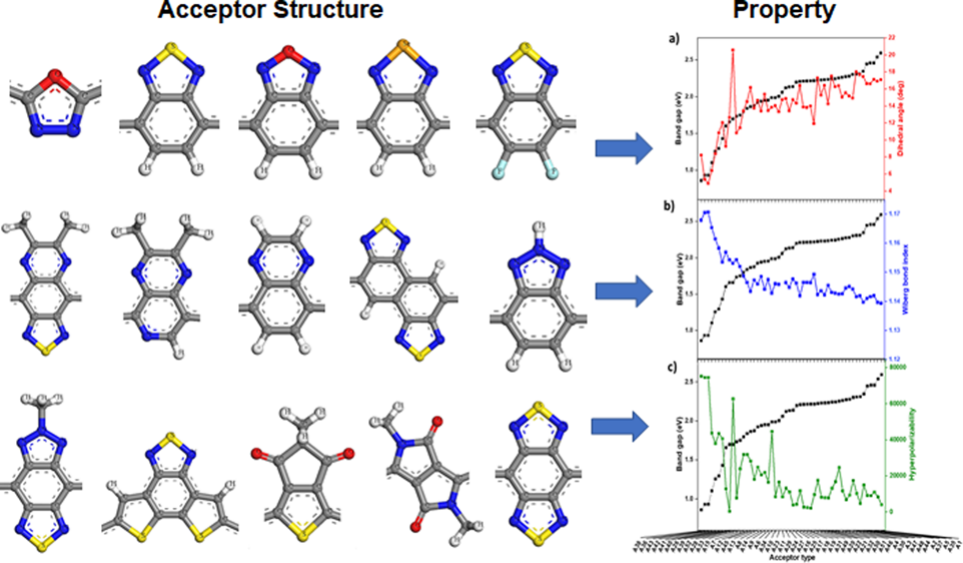

More than 50 different acceptor units from the experimental literature have been modeled, analyzed, and compared by using the computationally extracted data from the density functional theory (DFT) perspective for tetramer structures in the form of (D–B–A–B)_4_ (D, donor; A, acceptor; B, bridge) with fixed donor and bridge units. Comparison of dihedral angle between acceptor, donor, and bridge units, bond order, and hyperpolarizability reveals that these three structural properties have a dominant effect on the frontier electronic energy levels of the acceptor units. Systematic investigation of the structural properties has demonstrated the band gap energy dependency of the acceptor units on the planarity, conjugation, and the electron delocalization. Substitution effect, morphological alternation, and insertion of π-electron deficient atoms in A unit have also an important role to determine physical properties of the donor–acceptor conjugated polymers. This benchmark study will be beneficial for the band gap engineering and molecular design of the donor–acceptor copolymers using different acceptor units for the organic electronic applications.

## Introduction

Over the past decades, research in π-conjugated systems, especially donor–acceptor conjugated copolymers (CPs), has yielded significant advances in the creation of organic functional materials for electronic applications, especially for organic photovoltaics.^1^ CPs have an extensive network of overlapping π-orbitals throughout the whole polymeric backbone and, consequently, display distinctive optical and electronic properties.^2^ In addition, CPs possess important advantages toward inorganic semiconductors and small molecules such as low cost, ease at processing, lightweight, and flexibility.^3^ These properties make CPs particularly intriguing candidates for a wide range of optoelectronic applications such as organic light-emitting diodes (OLEDs), organic electrochromic (OECs), organic field effect transistors (OFETs), and organic photovoltaics (OPVs).^4−12^

Engineering of materials for organic electronics requires that they be designed for enhanced optical and electronic properties of CPs which can be effectively tuned by tailoring the proper electron-donating (donor, D) and electron-withdrawing (acceptor, A) building block units of resulting CPs. Especially, selection of D–A moieties enables control of the highest occupied molecular orbital (HOMO) energy and lowest unoccupied molecular orbital (LUMO) energy and thus the band gap energy (difference between HOMO and LUMO). Determination of the HOMO–LUMO energy level has an importance in terms of the optimized charge injection into and charge extraction out of the conducting polymer layer.^5,13^ Moreover, band gap energy shows the absorption edge which is an important parameter for the optoelectronic application of conducting polymers.

Compounds are identified as follows: **A1**, 1,3,4-oxadiazole;^14^**A2**, 1,3,4-thiadiazole;^15^**A3**, 3,6-dihydro-1,2,4,5-tetrazine;^15^**A4**, thiazolo[5,4-*d*]thiazole;^16^**A5**, 2,2′-bithiazole;^17^**A6**, benzo[*c*][1,2,5]thiadiazole;^18^**A7**, [1,2,5]thiadiazolo[3,4-*c*]pyridine;^19^**A8**, 5-fluorobenzo[*c*][1,2,5]thiadiazole;^20^**A9**, 5,6-difluorobenzo[*c*][1,2,5]thiadiazole;^21^**A10**, 5-ethoxybenzo[*c*][1,2,5]thiadiazole,^22^**A11**, 5,6-diethoxybenzo[*c*][1,2,5]thiadiazole;^22^**A12**, naphtho[1,2-*c*:5,6-*c*′]bis([1,2,5]thiadiazole);^23^**A13**, benzo[*c*][1,2,5]selenadiazole;^24^**A14**, benzo[*c*][1,2,5]oxadiazole;^25^**A15**, 2,2-dimethyl-2*H*-benzo[*d*]imidazole;^26^**A16**, 2*H*-benzo[*d*][1,2,3]triazole;^27^**A17**, 2-methyl-2*H*-benzo[*d*][1,2,3]triazole;^28^**A18**, 2-ethyl-2*H*-benzo[*d*][1,2,3]triazole;^28^**A19**, 2-propyl-2*H*-benzo[*d*][1,2,3]triazole;^28^**A20**, 5,6-difluoro-2-methyl-2*H*-benzo[*d*][1,2,3]triazole;^28^**A21**, 2,7-dimethyl-2,7-dihydronaphtho[1,2-*d*:5,6-*d*′]bis([1,2,3]triazole); **A22**, quinoxaline;^29,30^**A23**, pyrido[3,4-*b*]pyrazine;^31^**A24**, 2,3-dimethylpyrido[3,4-*b*]pyrazine;^31^**A25**, 2,3-difluoroquinoxaline;^32^**A26**, pyrazino[2,3-*g*]quinoxaline;^33^**A27**, 3*a*,4-dihydrothieno[3,4-*b*]thiophene; **A28**, 5,7-dihydrothieno[3,4-*b*]pyrazine;^34^**A29**, 5,5-dimethyl-4*H*-cyclopenta[*c*]thiophene-4,6(5*H*)-dione;^35^**A30**, 5-methyl-4*H*-thieno[3,4-*c*]pyrrole-4,6(5*H*)-dione;^36,37^**A31**, 5-methyl-4*H*-thieno[3,4-*c*]pyrrole-4,6(5*H*)-dione;^35^**A32**, naphtho[2,3-*c*]thiophene-4,9-dione;^35^**A33**, 2,5-dimethylpyrrolo[3,4-*c*]pyrrole-1,4(2*H*,5*H*)-dione;^38^**A34**, (*E*)-1,1′-dimethyl-[3,3′-biindolinylidene]-2,2′-dione;^39^**A35**, 2-methylisoindoline-1,3-dione;^40^**A36**, 3*a*,7*a*-dihydrobenzo[1,2-*d*:4,5-*d*′]bis(thiazole);^40^**A37**, 3*a*,7*a*-dihydrobenzo[1,2-*d*:4,5-*d*′]bis(thiazole);^41,42^**A38**, 6-methyl-6,8-dihydro-4*H*-[1,2,3]triazolo[4′,5′:4,5]benzo[1,2-*c*][1,2,5]oxadiazole; **A39**, 2,6-dimethyl-2,4,6,8-tetrahydrobenzo[1,2-*d*:4,5-*d*′]bis([1,2,3]triazole),^43^**A40**, 6,7-dimethyl[1,2,5]thiadiazolo[3,4-*g*]quinoxaline;^44^**A41**, 6-methyl-6,8-dihydro-4*H*-[1,2,3]triazolo[4′,5′:4,5]benzo[1,2-*c*][1,2,5]thiadiazole; **A42**, 6-methyl-6,8-dihydro-4*H*-[1,2,3]triazolo[4′,5′:4,5]benzo[1,2-*c*][1,2,5]selenadiazole; **A43**, 4*H*,8*H*-benzo[1,2-*c*:4,5-*c*′]bis([1,2,5]thiadiazole);^45^**A44**, 2,7-dimethylbenzo[*lmn*][3,8]phenanthroline-1,3,6,8(2*H*,7*H*)-tetraone;^46^**A45**, 5-methyl-6*a*,9*a*-dihydro-4*H*-thiazolo[4,5-*c*]thieno[2,3-*e*]azepine-4,6(5*H*)-dione; **A46**, 2,6-dimethyl[1,2,3]triazolo[4,5-*f*]isoindole-5,7(2*H*,6*H*)-dione; **A47**, dithieno[3′,2′:3,4;2″,3″:5,6]benzo[1,2-*c*][1,2,5]oxadiazole;^47^**A48**, dithieno[3′,2′:3,4;2″,3″:5,6]benzo[1,2-*c*][1,2,5]thiadiazole;^47^**A49**, 5-methyl-4*H*-dithieno[3,2-c:2′,3′-e]azepine-4,6(5*H*)-dione;^48^**A50**, 5-methyl-6*a*,9*a*-dihydro-4*H*-thiazolo[4,5-*c*]thieno[2,3-*e*]azepine-4,6(5*H*)-dione;^49^**A51**, 5-methyl-4*H*-dithiazolo[4,5-*c*:5′,4′-*e*]azepine-4,6(5*H*)-dione; **A52**, benzo[*lmn*][3,8]phenanthroline-1,3,6,8(2*H*,7*H*)-tetraone^50^

**Figure 1:**
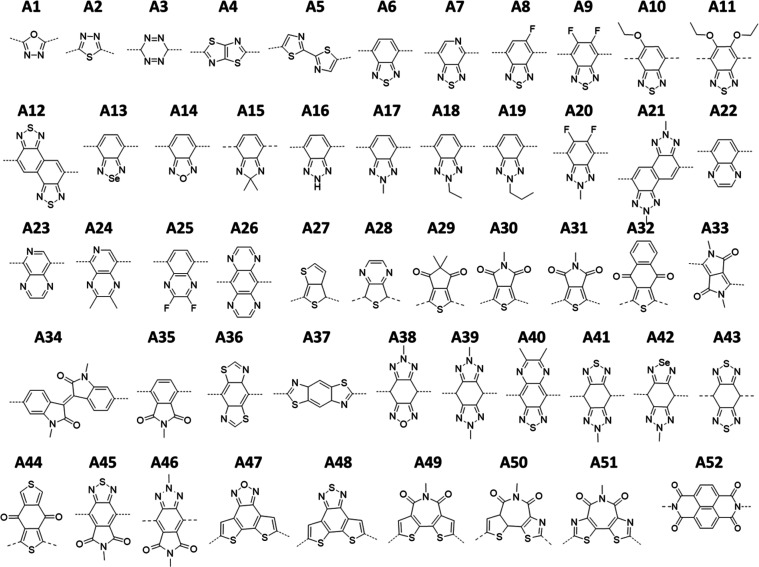
Representative structures of conjugated acceptor units utilized in this study.

Previous theoretical and experimental studies in the past decade presented the enhanced electronic properties of some of the most used D–A units.^2,51−56^ However, this is the first comprehensive computational study comparing and analyzing more than 50 acceptors, which have drawn most of the attention in both academic studies and industrial applications selected from the recent literature. Therefore, our study will give an insight into the relation of design principles and structural properties and help scientists find optimal performance conducting materials for the electronic applications. Systematic investigation of a series of acceptor moieties is achieved from geometrically optimized oligomers containing an alternating acceptor unit coupled with 4,8-bis(5-methylthiophen-2-yl)benzo[1,2-*b*:4,5-*b*′]dithiophene (PBDTT-BT) as a donor and thiophene as a bridge (B) unit, while maintaining the same tetramer conjugated chain oligomer structure. Determination of acceptor units, shown in Figure 1, is based on the most used structures in the OPV, OLED, OFET, and OEC applications in the literature. Electrical and optical band energy gaps, electrostatic potential surface (ESP), HOMO and LUMO energy levels, bond order calculated from Wiberg bond index (WBI), planarity, polarity, polarizability, and hyperpolarizability of the coupled conjugated system are predicted using data computationally extracted from the perspective of density functional theory (DFT). In addition, reorganization energies (λ_reorg_) and excited-state vertical and adiabatic transition of the oligomers were calculated to predict mobility of the model structures.

## Theoretical Calculations

Theoretical calculations were performed by using Gaussian09 (A02 software package).^57^ Density functional theory (DFT) methods were applied for the tetramers, in the form of (D–B–A–B)_4_ (D, donor; A, acceptor; B, bridge) at B3LYP hybrid functional with 6-311g(d) basis set with tight SCF convergence criteria which have presented successful results in previous studies.^58−60^ Geometry optimizations were started from different initial conformations such as the relative conformation of donor and acceptor by controlling the torsional angle between connected donor, acceptor, and bridge units, as shown in Figure S1 systemically to determine the lowest energy geometries. Electrostatic potential surface (ESP), highest occupied molecular orbitals (HOMO), and lowest unoccupied molecular orbitals (LUMO) were calculated for the optimized geometries of tetramers. Dihedral angle (θ) between the bridge and donor and between donor and bridge were calculated for the corresponding bonds which are represented as number and shown in Supporting Information Figure S1. Band gap was calculated by using two different methods that are the direct difference between the HOMO energy and the LUMO energy for the optimized ground state and the calculation vertical excitation energy of the lowest singlet excited state (S_0_ → S_1_). The singlet excited states of the oligomers were calculated by using time-dependent density functional theory (TDDFT) at the same level of calculation quality. Vertical ionization potential (VIP) and adiabatic ionization potential (AIP) were calculated by the energy difference between the neutral tetramer and cation state of the optimized ground state geometry, followed by optimized cation geometry, respectively. Hole reorganization energies (λ_reorg_) were determined on the basis of the formulation by Marcus and by Bredas et al.^61,62^ Charge transfer between acceptor and donor were calculated on the basis of the ESP fitting scheme of Merz–Singh–Kollman (MK) and natural population analysis (NPA) at the same level of theory. Wiberg bond indexes were calculated using NPA method for the for the corresponding bonds, which are represented as letters and shown in Figure S1. Orbital composition analysis was performed by using Multiwfn wave function analysis program in three partition schemes, Mulliken, Stout–Politzer, and Ros–Schuit.^63^

## Results and Discussion

For comparison of the organoelectronic performances of acceptor units as shown in Figure 1, the donor (D) and bridging (B) units were kept the same for allof the tetramers. 4,8-Bis(5-methylthiophen-2-yl)benzo[1,2-*b*:4,5-*b*′]dithiophene (PBDTT-BT) is chosen as the donor unit for all of the tetramers, since benzo[1,2-*b*:4,5-*b*′]dithiophene (BDT) and its derivatives have been widely used among the electron donating monomers in the literature owing to its planar and symmetric structure and high charge transport ability.^64^ Next, thiophene is used as a π-bridge unit as it has a relatively small and electron rich structure that provides absorption of longer wavelengths in the spectrum with weaker steric hindrance.^65^

The control of the HOMO–LUMO levels and therefore the band gap engineering of the conjugated systems has the major importance in the design of the corresponding materials for the optoelectronic applications. In the literature, the major driving force on control of the band gap of extended π-conjugated systems have been studied for some donor–acceptor structures for years.^66^ For the comparison of the band gap and for elucidating the structural and electronic characteristics affecting the frontier orbital energy levels, two different methods were used. First, HOMO and LUMO energy levels were calculated, and taking that the difference between these energy levels on the optimized ground state gives the band gap, the second method is the calculation of the vertical excitation energy of the lowest singlet excited state (S_0_ → S_1_).

**Figure 2:**
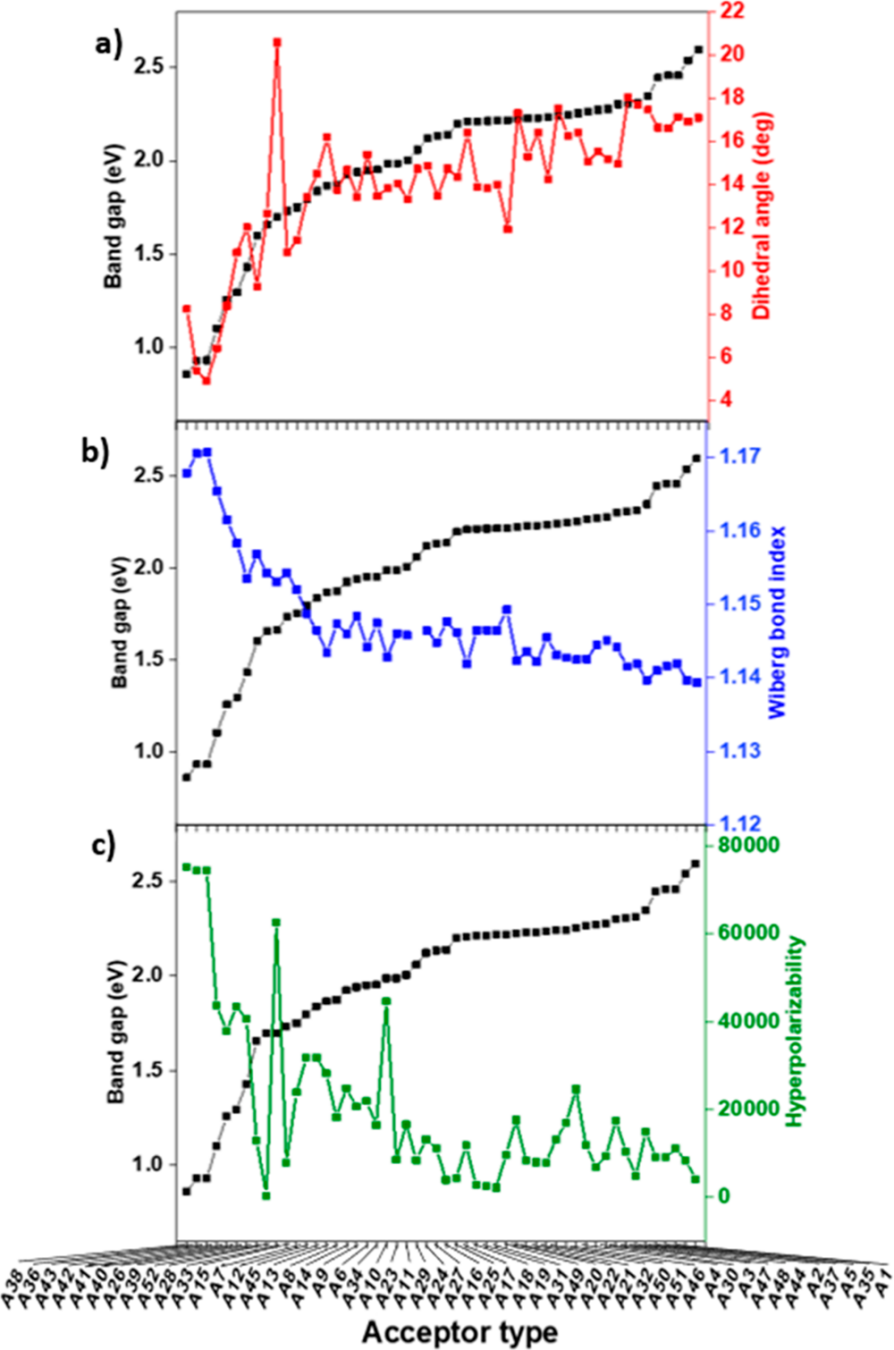
Effects of structural properties on the band gap energy for 52 tetramers with different acceptor units. (a) Dihedral angle, (b) Wiberg bond index, and (c) hyperpolarizability effect on the band gap energy.

Previous studies show that the planarity of the conjugated systems has a significant contribution to the band gap, since the dihedral angle between the units in the polymers limits the inter-ring rotations and the delocalization of the π-electron.^66^ Dihedral angles between the bridge and alternating acceptor units and between the bridge unit (thiophene) and fixed donor unit (4,8-bis(5-methylthiophen-2-yl)benzo[1,2-*b*:4,5-*b*′]dithiophene) were measured for optimized geometries and given in Table 1 and Table S2. In a general trend, it can be deduced that the dihedral angle between bridge and the donor unit has more significant impact on the HOMO–LUMO band gap whereas covalent rigidification between the bridge units and the alternating acceptor unit does not have influence on the band gap. HOMO–LUMO band gaps of the molecules tend to decrease with improved planarity characteristics of π-conjugated path resulting in weak electron hopping across the backbone, as shown in Figure 2a. Although the same donor and bridge units were used for all tetramers, the dihedral angle between the donor and bridge units has changed with different acceptor units. This indicates that alternating acceptor type has an influence on the dihedral angle between the bridging unit and the donor; therefore it has a role on the control of band gap energy in the tetramers.

**Table 1:** Structural and Electronic Properties of the Oligomers with 10 Selected Acceptors^a^

acceptor	*E*_band gap_ (eV)	λ_reorg_ (eV)	μ (debye)	θ_(D–B)_	θ_(A–B)_
A6	1.95	0.09	0.31	13.47	6.25
A12	1.84	0.08	0.36	14.51	6.3
A14	1.94	0.09	2.81	13.43	1.96
A16	1.87	0.09	1.62	14.34	2.29
A22	2.23	0.09	1.27	16.4	23.33
A28	1.66	0.1	0.6	12.66	0.71
A30	2.28	0.09	2.68	15.18	1.95
A39	1.6	0.09	0.9	9.29	0.49
A43	0.93	0.16	0.92	4.91	0.33
A48	2.31	0.09	2.44	17.68	19.11

a Parameters of the other acceptors are given in the Supporting Information.

Next, the Wiberg bond indices (WBI) between the donor and bridge units and between the acceptor and bridge units were calculated by excluding the two end groups to prevent the end group effects. WBI have been used to measure the bond order, leading us to the information on the π-bond character and conjugation on the backbone.^67^ For the bond index higher than 1.0, it can be interpreted as there is more than one pair of electrons shared between them. WBIs of corresponding bonds for tetramers were found in the range changing from 1.13 to 1.17, resulting in all of the acceptor units contributing the π-conjugation property and increasing the strength of bonds and shortening the bond lengths. Moreover, results showed that WBI between the donor and bridge units decreases with increasing band gap energy since insertion of double bonds leads to a decrease of the overall aromaticity of the system and thus to a further reduction in band gap, as shown in Figure 2b. The highest WBI is 1.17 for a derivative of thiazole (**A36**) having 0.93 eV band gap energy. Like the dihedral angle between the bridge unit and acceptor units, the WBI index between them has also not changed as changing acceptor units. It can be concluded that the tailored acceptor units have a major role and control on the structure in a sense of the changing position and geometry of the donor units, resulting in the change in planarity and conjugation of the tetramers.

Another structural effect on the band gap is the first order hyperpolarizability of the polymers, which is given in Figure 2c. In the literature, it is directly proportional with the reduction of bond length alternation (BLA) in donor–acceptor conducting polymers.^68^ The dependence of hyperpolarizability on the bond length alternation has been shown with enhanced electron delocalization.^69^ Monomers of the acceptor units in the form of BDBAB were constructed, and optimized geometries were used for the calculation of the hyperpolarizability. Results demonstrate that there is a decreasing trend for the acceptor unit within an increased band gap energy, which is in parallel with preliminary studies for π-conjugated compounds.

Effect of the alkyl groups of the acceptor units on the band gap energy was locally observed among the tetramers. Benzotriazole derivatives were substituted with hydrogen, -methyl, -ethyl, and -propyl for **A16**, **A17**, **A18**, and **A19**, respectively. With increasing of the steric hindrance in the alkyl group, the band gap energies of the tetramers also increase with an almost the same dihedral angle between the acceptor and bridge units. Substitution of the longer alkyl chains has advantages for the dissolving of the polymer in organic solutions for the film preparation for organic electronic applications; however, it causes a slight increase in the band gap energy that may hinder the efficiency of the device. However, the type and number of the substituents have an effect on the dihedral angle between the bridge and acceptor units. An additional two -methyl substitutions on the derivative of pyrazine promote an increase in the dihedral angle between the acceptor and bridge units on the tetramer from 9.6 to 11.9°, as can be observed in comparison of **A23** and **A24**. The same trend was also observed for the -ethoxy substituent on the derivatives of the benzothiadiazole for **A10** and **A11**. As shown in Table S2, two -ethoxy substitutions for **A11** cause a 12.76° torsion between the acceptor and bridge units, while the corresponding dihedral angle of **A10** is 7.24° with more planar conformation for the tetramer. Also, the **A10** tetramer presents a helical structure, while the tetramer of **A11** substituted by two groups has a more planar structure due to the lower dihedral angle. In addition, doubling the substituent creates symmetry and planarity, leading to the dipole moment (μ) of **A11** being higher (2.02) than the dipole moment of **A10** (1.54). Branched alkyl substituents such as 2-ethylhexyl, which are also widely used in these systems to enhance organic solubility and film morphologies, can decrease the planarity and alter the band gap slightly that was calculated as a 0.02 eV decrease in the band gap and 5° increase in the dihedral angle for **A17** by 2-ethylhexyl substitution.

Comparison of ESPs for **A11** and **A10** shows single substitution of the -ethoxy group makes the donor unit less positive, which can be also observed from the natural population analysis (NPA) charges of the units, which are given in Table S2. However, fluorine atom substitution creates a different pattern for the planarity of the tetramer. **A8** and **A9**, which are the derivatives of the benzothiadiazole with one and two fluorine atoms, have the dihedral angle between the acceptor and bridge unit 2.3 and 0.8°, respectively, while the dihedral angle between the donor and the bridge of them was almost the same. Substitution of the second fluorine substituent increases the band gap energy from 1.92 to 1.95 eV with no change in dipole moment for the tetramers. Investigation of **A17** and **A20** showed that the alternation of two hydrogen atoms on the derivative of benzotriazole with fluorine has almost no effect on the dihedral angle or band gap energy; however, the dipole moment of the molecule has changed from 2.5 to 4.3. In addition, the same trend was also observed in a different type of acceptor, **A22** and **A25** as shown in Table 1 and Table S2. By this way, deviations in the electronic characteristics of tetramers via control of the substituent type on the acceptor units were elucidated.

The bonding point has a role on the electronic character of the tetramers also. To investigate this behavior, the same acceptor unit with two different attaching points between the acceptor and bridge units were compared with **A36** and **A37.** A benzobis(thiazole) derivative is attached to the bridging unit from the benzene ring for **A36**, whereas the thiazole part is used for the attachment for **A37**. This positional alteration creates a dramatic increase in the dihedral angle between the donor and bridging units, in addition to a slight increase in the angle between the acceptor and bridging unit. As a result of the lowered planarity and shorter conjugation length of the tetramer, an energetically more stable form of the tetramer was constructed with the band gap energy increased from 0.7 to 2.1 eV as the position changed from the vertical to horizantal. Besides, from the dramatic increase in the band gap, WBI between donor and bridge units and acceptor and bridge units decreases, leading to a reduction in the electron delocalization of the tetramer. In addition to the alternation of substitution atom on acceptor, the change of the position of the acceptor units in the same tetramer was also examined. **A30** and **A31** were prepared by taking into account the position of the sulfur atom in the acceptor unit with respect to the sulfur atom in the thiophene as bridging unit. The tetramer of **A31** is designed as the thiophene part of the acceptor unit and is in the same directional orientation with two bridging thiophenes; this brings three sulfur atoms in a close position with respect to **A31**. This orientational change also creates electrostatic and dipole–dipole interaction between the oxygen atom of the acceptor and the hydrogen atom of the thiophene on the neighboring acceptor in **A31**. For the **A30** tetramer, the carbonyl oxygen creates a repulsion with the sulfur of the bridge unit. That is the reason that the energetically more stable form, **A31**, shows a lower band gap energy (2.22 eV) and better planarity with lower hyperpolarizability. It can be concluded that, in addition to the electronic properties of the tetramers, the direction of the acceptor units has a significant role on the morphology of the donor–acceptor conducting polymers.

The importance of comparison for oxygen, sulfur, and selenium substitutions in different acceptor units which have the same molecular structure for the rest of the system have been underlined and studied in literature.^70,71^ Here, the comparison of oxygen, sulfur, and selenium was shown using benzochalcogens shown with **A14**, **A6**, and **A13**, respectively. Contrary to the previous study, results show that selenium substitution instead of the oxygen has a significant role on the planarity of the tetramer.^71^ The dihedral angle between the acceptor and bridge unit is 11.1° for **A13** and 1.9° for **A14**, whereas **A6** has 6.25° as dihedral angle with sulfur substitution. Increasing the atomic size in the substituent atom leads to a higher deviation from the planarity of the tetramers affecting the intermolecular charge transfer between the acceptor and donor unit. NPA-based charge transfer for tetramers gave 0.16, 0.11, and 0.10 for **A14**, **A6**, and **A13**, respectively. As represented in Table 1 and Table S3, reorganization energy of **A14**, which is an important parameter for the organic electronic device efficiency, is the lowest in this comparison, leading the higher efficiency potential for OPV application as a result of higher charge transfer and charge mobility. To set more general rules about oxygen, sulfur, and selenium containing units, larger and smaller acceptor units were investigated to exclude the acceptor unit size effect. The trend for the enhanced planarity for the smaller chalcogen substitution were validated for the second and third sets of acceptor units with different substitutions, **A38**, **A41**, and **A42** for oxadiazole, thiadiazole, or selenodiazole ring comparison in relatively larger structures, in addition to the **A1** and **A2** for oxadiazole and thiadiazole in relatively smaller acceptor unit structures. In addition, comparison of **A47** and **A48** showed that the increase in the number of aromatic rings does not have a significant role on the reorganization energy of the donor–acceptor polymers as shown in Table 1 and Table S6.

**Figure 3:**
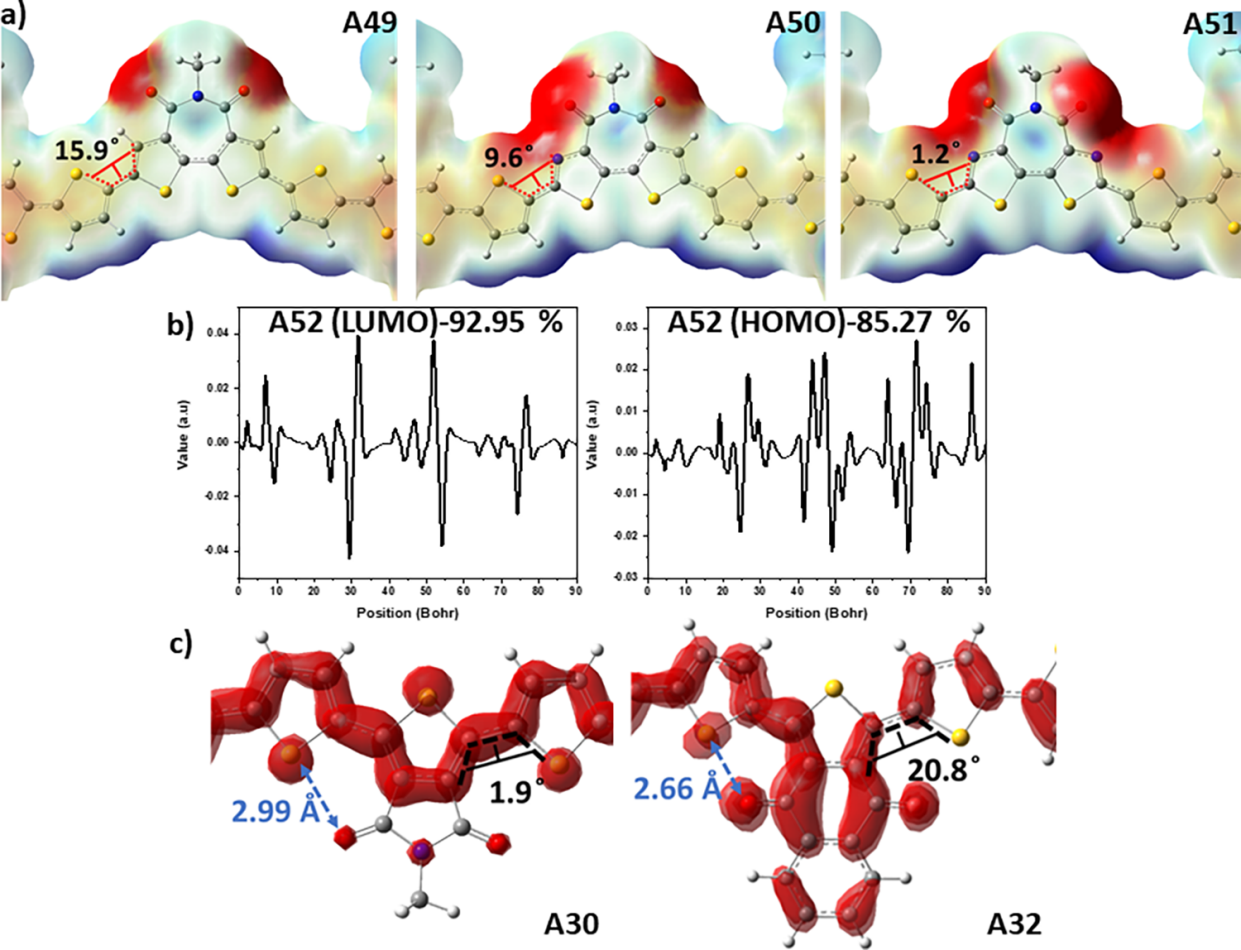
(a) ESP surface of the A49, A50, and A51. (b) LUMO and HOMO distributions on the acceptor and donor of the A52. (c) Overlappings of the HOMO and LUMO frontier orbitals of A30 and A32.

The impact of the insertion of π-electron deficient atoms was investigated by replacement of sp^2^ carbon atom by a more electronegative imine nitrogen atom in the aromatic ring of the acceptor units. **A7**, having the only structural difference from **A6** as the nitrogen atom on the pyridine ring, showed a higher vertical electron affinity (VEA), and the higher charge transfer between the acceptor and donor units. The higher electronegativity of nitrogen atom and its incorporation into the polymeric backbone provides a lower band gap energy, deeper-positioned HOMO–LUMO energy levels, resulting in a better, stronger acceptor unit for the organic electronic applications as reported previously.^49^ Besides, the pyridine ring fused to the thiadiazol ring; the same trends were also observed for the comparison of quinoxaline (**A22**) with **A23** with the replacement of a carbon atom by nitrogen atom. The comparison of the **A49**, **A50**, and **A51** showed that the second additional electron deficient atom elevates the acceptor strength of the units even for large fused systems. Tetramers containing imide-functionalized arene structures are affected from this replacement, keeping the band gap energy almost the same (∼2.22 eV), with an enhanced planarity of the backbone due to the decreasing dihedral angle between the bridge and acceptor unit (15.9°, 9.6°, and 1.2°, respectively). This observation can be explained by the stronger intramolecular noncovalent interactions between the sulfur atom in the bridge and the nitrogen atoms on both sides of the acceptor unit on **A51** that promotes a backbone planarity and transfer of electron density as shown in ESP images of the **A49**, **A50**, and **A51** in Figure 3a.

Fusion of heteroatomic aromatic rings with a higher resonance energy have been used in the literature to increase the quinoid character of the neutral state for the donor–acceptor conjugated polymers.^66^ To investigate the effect of the fused electron accepting units, tetramers containing acceptor units with fused rings, **A12** and **A21**, were compared with the tetramers which contain their nonfused counterparts, **A6** and **A17**, respectively. The presence of fused ring acceptor units from the benzene ring with thiazole and methyl triazole ring units in tetramers elevates the hyperpolarizability about two times, whereas the planarity of the tetramers slightly decreased for both **A12** and **A21**. Fusion of the rings decreased the molecular hole reorganization energy from 0.09 to 0.08 for both **A6** and **A12** and for **A17** and **A21**, resulting in the enhanced both intra- and intermolecular charge hopping, thereby the increasing charge carrier mobility as shown in Table 1 and Table S6. Total atomic charge on the donor and acceptor units measured by the NPA analysis also presents that the intermolecular charge transfer between the donor and the acceptor units also increased with the insertion of the fused acceptor units into the donor–acceptor conjugated polymer systems as shown in Table S2. The same enhancement for the two different sets for the acceptor units shows that increasing aromaticity of the system by fusing the aromatic structures is independent from the type of the acceptor units. Due to these improved electronic properties, the acceptor units with fused aromatic structures attracting more attention in the recent literature of organic solar cell applications. In addition to these, **A4** and **A5** that contain the derivative of the thiazolothiazole and bithiazole, respectively, reveal another perspective about the fusion of the units. **A4** has a fused ring, which has a rigid planar structure, and **A5** has two thiazole rings, which are bonded to each other with a single bond. Contrary to the previous fused systems, **A5** yields hyperpolarizability almost two times higher than **A4**, resulting in the higher band gap energy. The planarity of **A5** is lower than the planarity of **A4**, even though the dihedral angle between the thiazole rings in **A5** is close to 180°. Although it has decreased planarity and higher band gap, **A5** has a lower reorganization energy than that of **A4**, probably due to the extended interchain π-conjugation along the polymeric backbone.^17^ Another fused system is a derivative of an isoindigo unit, **A34**, which has been especially widely used in the electrochromism due to the huge π-system and yielded 1.99 eV band gap energy with a moderate planarity for the tetramer resulting in 13.84° as dihedral angle between the donor and the bridge unit. Among all teramers, polycyclic molecules such as **A38**, **A36**, and **A43** has the lowest band gap energies, lower than 1.0 eV, which can be potential candidates for the OPV applications. On the contrary, tetramers have acceptor units of **A1** and **A35**, having band gap energies higher than 2.5 eV and acting as potential acceptor units for the OLED applications.

**Figure 4:**
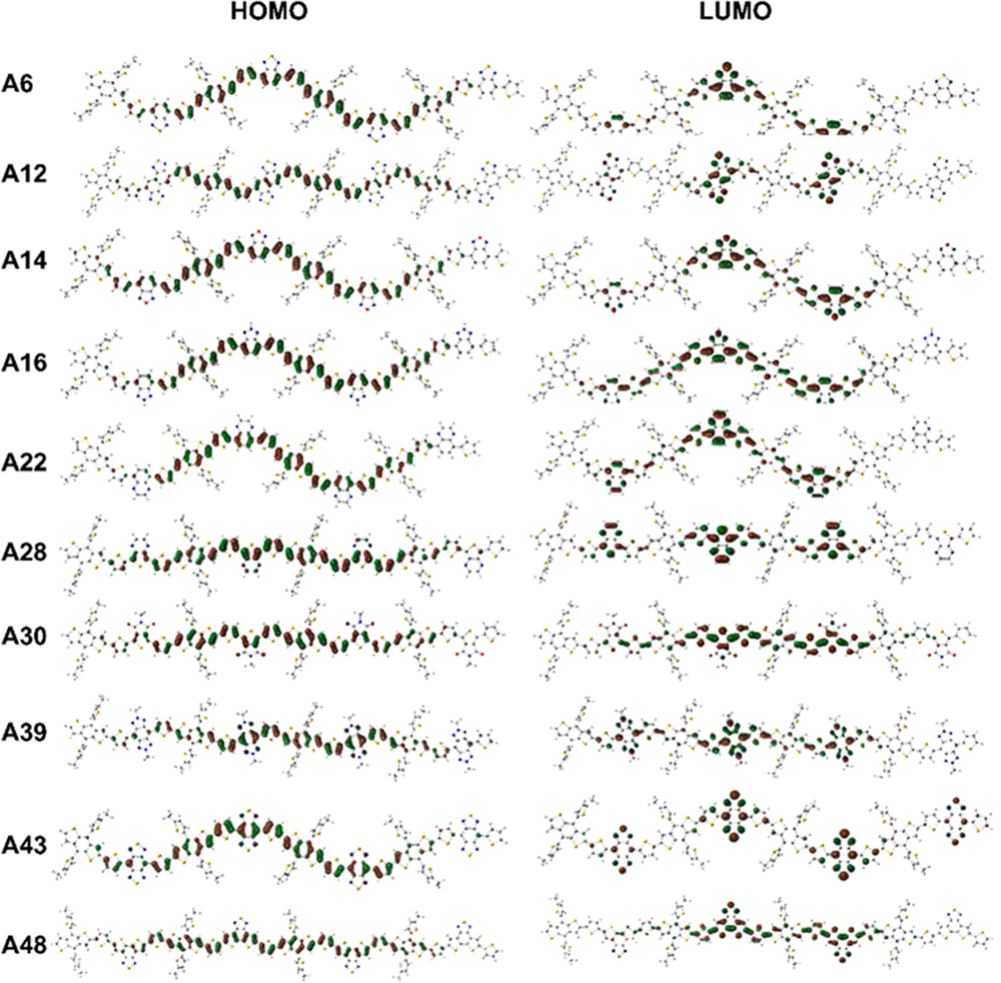
HOMO and LUMO isosurfaces of 10 selected tetramers with different acceptor units.

Molecular orbital surfaces of HOMO and LUMO for the optimized geometries of tetramers of ten selected tetramers with different acceptor units were given in Figure 4. Frontier orbitals of 52 tetramers that are utilized in this study can be found in Figure S2. As a general trend, HOMO orbitals were delocalized along the chain for tetramers instead for localizing on the donor–acceptor units. LUMO orbitals were also delocalized, however less than HOMO, where LUMO orbitals are still mostly placed on the acceptor units as expected. Comparison of **A39** and **A43** shows the LUMO orbitals localization on the acceptor units as the elements in acceptor units become more electronegative. The percentage distribution of LUMO on the acceptor was changed from 53 to 71% when a sulfur atom is replaced with the nitrogen atom in the acceptor unit with respect to the Mulliken orbital composition analysis results. As shown in Table S4, the distribution of the molecular orbitals was calculated using three partition schemes, Mulliken, Stout–Politzer, and Ros–Schuit, and results of the three analysis methods are close to each other for almost all tetramers. Localization of LUMO on acceptor units by changing the electronegativity of the substituent atoms in the acceptor unit can also be observed for the comparison of **A6**, **A8**, and **A9**. For benzothiadiazole (**A6**) the distribution percentage of the LUMO on the acceptor unit is 60 by Mulliken orbital composition analysis, and it rises to 62 and 64 when hydrogen atom is changed with one and two fluorine atoms as substituent, respectively. One of the highest differences in the percentage distribution belongs to **A52** with the band gap energy as 1.70 eV and the HOMO composition is on donor units by 92.95, 91.71, and 91.13%, whereas the LUMO distribution is positioned on the acceptor units by 85.27, 85.54, and 82.79% for Mulliken, Stout–Politzer, and Ros–Schuit, respectively. The repetitive pattern in the distribution of HOMO and LUMO on the tetramer with a higher localization percentage of frontier orbitals on acceptor and donor units are given for **A52** in Figure 3b. This type of frontier orbital distribution provides an enhanced intramolecular charge transfer resulting in improved experimentally observed photovoltaic device performance for **A52**.^72^ In Figure 3c, the HOMO and LUMO frontier orbitals are changing with a change in the position of the substitutional group to the thiophene group as acceptor unit. **A30** and **A32** both lead to an interaction between the sulfur atom on the thiophene as bridging unit and carbonyl oxygen of the acceptor unit. The distances of thienyl sulfur to carbonyl oxygen were calculated as 2.99 and 2.66 Å for **A30** and **A32**, respectively. The decrease in the distance between the thienyl sulfur and carbonyl oxygen provides an enhancement in the overlapping of the HOMO and LUMO orbitals on the conjugated backbone for **A32**. The dihedral angles between the acceptor and the bridge unit were calculated as 1.9 and 20.8° for **A30** and **A32**, respectively. The lowered planarity indicates that the increasing interaction between thienyl sulfur and carbonyl oxygen as well as the overlapping of the orbitals on substitutional groups in the polymeric backbone as a result of the conformational change in the carbonyl oxygen in acceptor units. The same trend can also be observed for the comparison of **A29** and **A44**.

**Figure 5:**
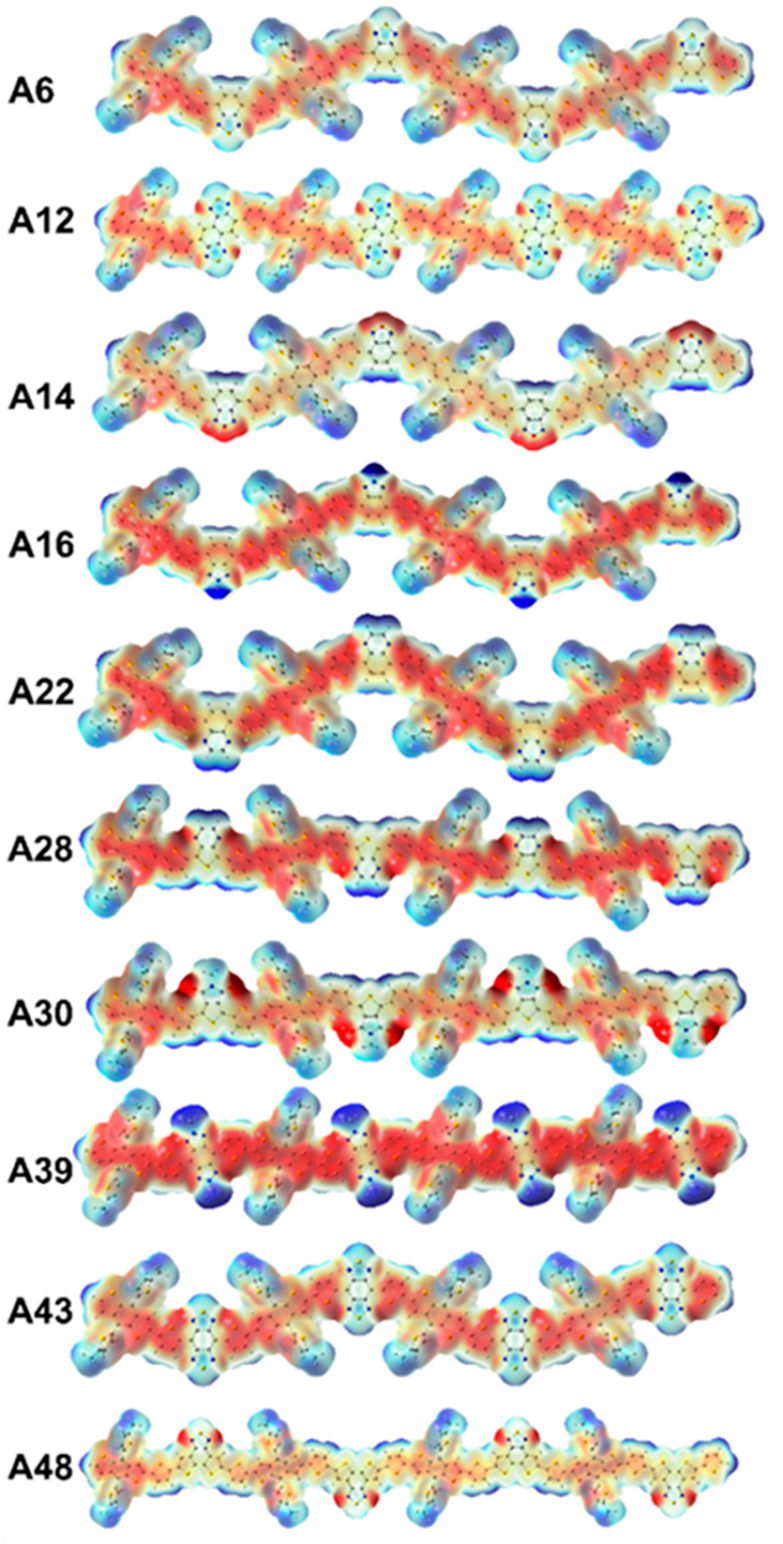
ESP surface of 10 selected tetramers with different acceptor units.

ESP surfaces of the molecular orbitals were given in Figure 5, showing well-ordered and sequential distribution of electron rich (red) donor and electron deficient (blue) acceptor sites given for the 10 selected tetramers out of 52. ESP surfaces for all of the tetramers modeled in this study can be found in Figure S3. Localization of the electron deficient part on the acceptor units represented by the blue color in the electrostatic potential surface leads to the better acceptor properties. Thus, the enhancement of the charge transfer between the different acceptor units and the fixed donor unit has emerged as one of the methods for convenient HOMO–LUMO engineering for the donor–acceptor polymers. Higher localization of the electron deficient part on the acceptor units and electron rich part on the donor unit can be defined as an indication for the strength of the donor or acceptor units related to the excess of the intramolecular charge transfer between these units. The total average atomic charges by ESP and NPA charges on one acceptor unit and one donor unit in the middle of the tetramers were calculated by neglecting end group donor–acceptor units to avoid end group effect. Results showed that the NPA charges were more convenient for comparing the charge transfer between the donor and acceptor units, since almost all of the acceptor units in the tetramers showed electron deficient behavior, and most of the donor units have positive NPA charges, as expected. The reason for the NPA charges gives more convenient results than the NBO method, which is an orbital-dependent technique that is based on the method for optimally transforming a given wave function into localized form, corresponding to the center of the atom.^73^ Atomic charges based on ESP fitting, on the other hand, were not properly distributed atomic charges since it is based on the molecular electrostatic potential fitting with respect to the atom radius in a system with several neighboring electron rich and deficient sites around the molecule. Calculated grid points are located on some layers around the molecule, and these layers are constructed as an overlap of van der Waals spheres around each atom with different electronic characters.^74^ This shows ESP charges on the corresponding atoms can be affected by the other atoms on the tetramer and result in weaker charge distribution. In addition to that, the ESP method is highly functional and basis set dependent, leading to the deviation of results with different advanced functional and basis sets. To investigate the origin of this observation, the monomer of the acceptors with fixed donor and bridge units is also constructed in BABD structural form. ESP charge distribution for the monomers was given in the Table S3.

## Conclusions

The most widely used 52 acceptor units in the donor–acceptor conjugated polymers systems were investigated to determine general rules for the electronic and structural properties using first principles calculations. The same functional and basis sets were used for structural optimization and further calculations for the first time on the many acceptors that were prepared by the same way to provide more comparable results between them. The generalization of the results of the different structures of the acceptors showed that the band gap energy has a direct relation with the planarity of the backbone, bond index between D–B–A and the hyperpolarizability of the chains among the alternating acceptor units with fixed donor and bridge units. The optimized structures, HOMO and LUMO frontier orbital levels, the distribution of these frontier orbitals, and reorganization energies of the tetramers can be tailored for the donor–acceptor conjugated polymers by the control of acceptor group for the engineering of structure and properties for the organic electronic applications.

## Supporting Information Available

HOMO, HOMO–1, LUMO, and LUMO+1 frontier orbitals; ESP; dihedral angles; band gap energy; optical band gap energy; dipole moment; polarizability; anisotropic polarizability; hyperpolarizability; VIP; AIP; VEA; reorganization energies; NPA charges of units; HOMO and LUMO distribution percentages (PDF)
Geometry optimized structures for the (D–B–A–B)_4_ (D, donor; A, acceptor; B, bridge) tetramers (ZIP)
