# Supplementary material for: Design Principles for the Acceptor Units in Donor–Acceptor Conjugated Polymers
Source: ACS Omega. 2022 Oct 18;7(43):38969–78. doi: 10.1021/acsomega.2c04713 (PMC9631416; doi:10.1021/acsomega.2c04713)
Supplement: Supplementary file 1 — ao2c04713_si_001.pdf [file ao2c04713_si_001.pdf]

Supporting Information

Design Principles for the Acceptor Units in Donor–Acceptor Conjugated Polymers

Tuğba Hacıefendioğlu,[†] *and Erol Yıldırım*[*, †, ‡, §]

[†]*Department of Chemistry, Middle East Technical University, 06800, Ankara, Turkey.*

[‡]*Department of Polymer Science and Technology, Middle East Technical University, 06800, Ankara, Turkey.*

[§]*Department of Micro and Nanotechnology, Middle East Technical University, 06800, Ankara, Turkey.*

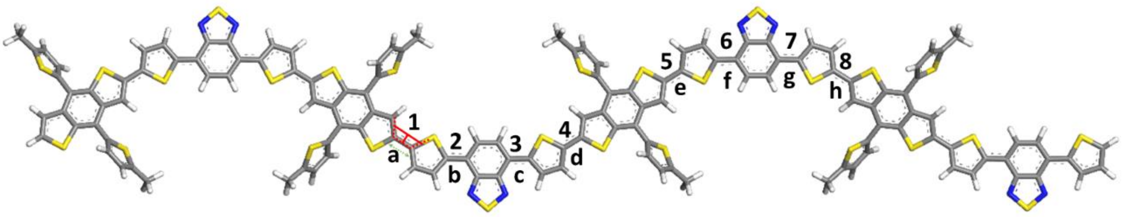

Figure S1 Representation of the dihedral angles (represented as numbers), length and bond index (represented as letters). Dihedral angles which are labelled as a, d, e and h shows the torsion angle between the donor and bridge unit, whereas b, c, f and g represent the torsion angle between the acceptor units and bridging unit. Numeric values of the dihedral angles are 14.37, 12.39, 12.61 and 14.48 for a, d, e and h and 7.73, 7.99, 4.25, and 5.05 for b, c, f and g.

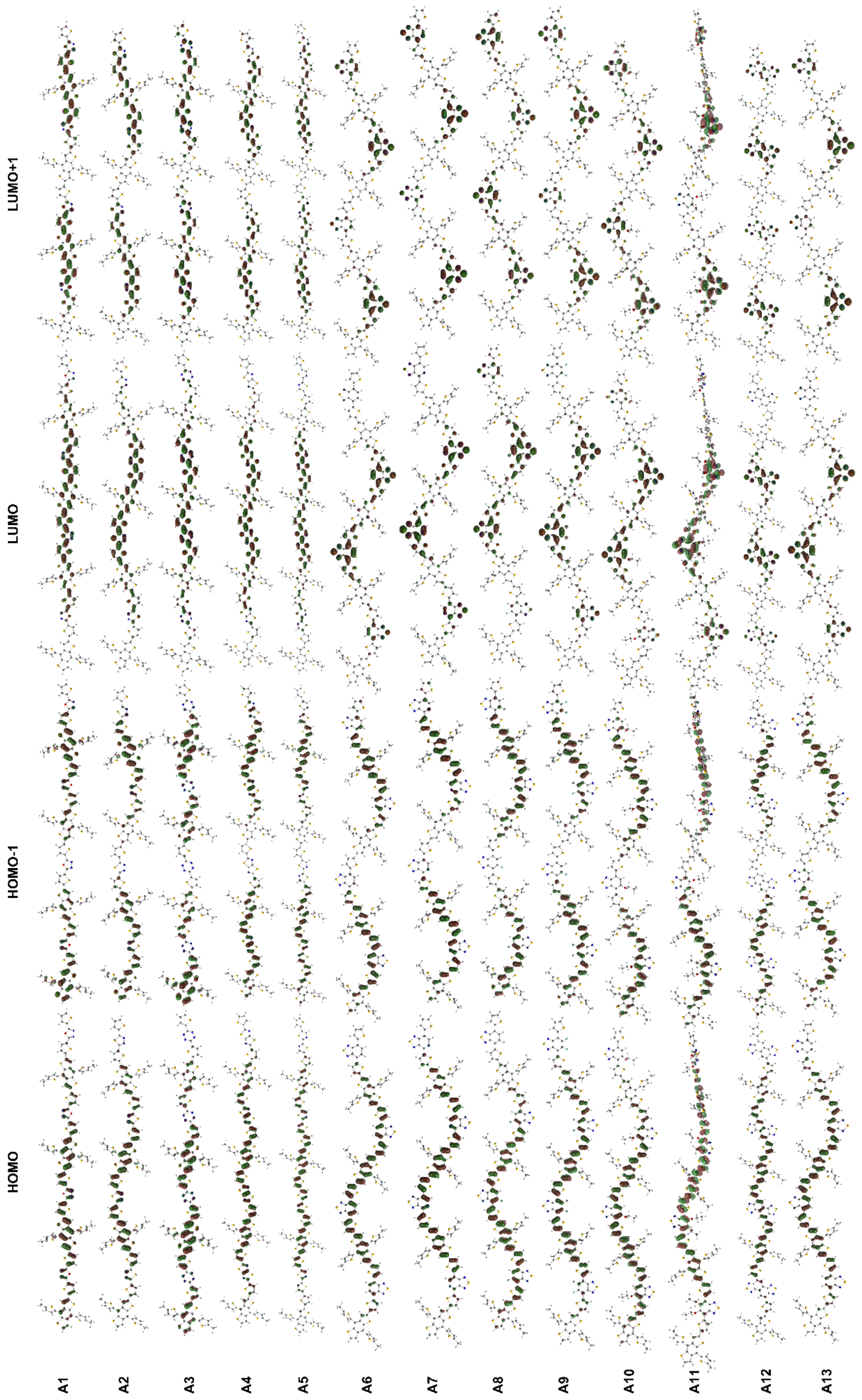

Figure S2 HOMO, HOMO-1, LUMO, LUMO+1 frontier orbitals of the tetramers of the 52 acceptor units.

Continue Figure S2

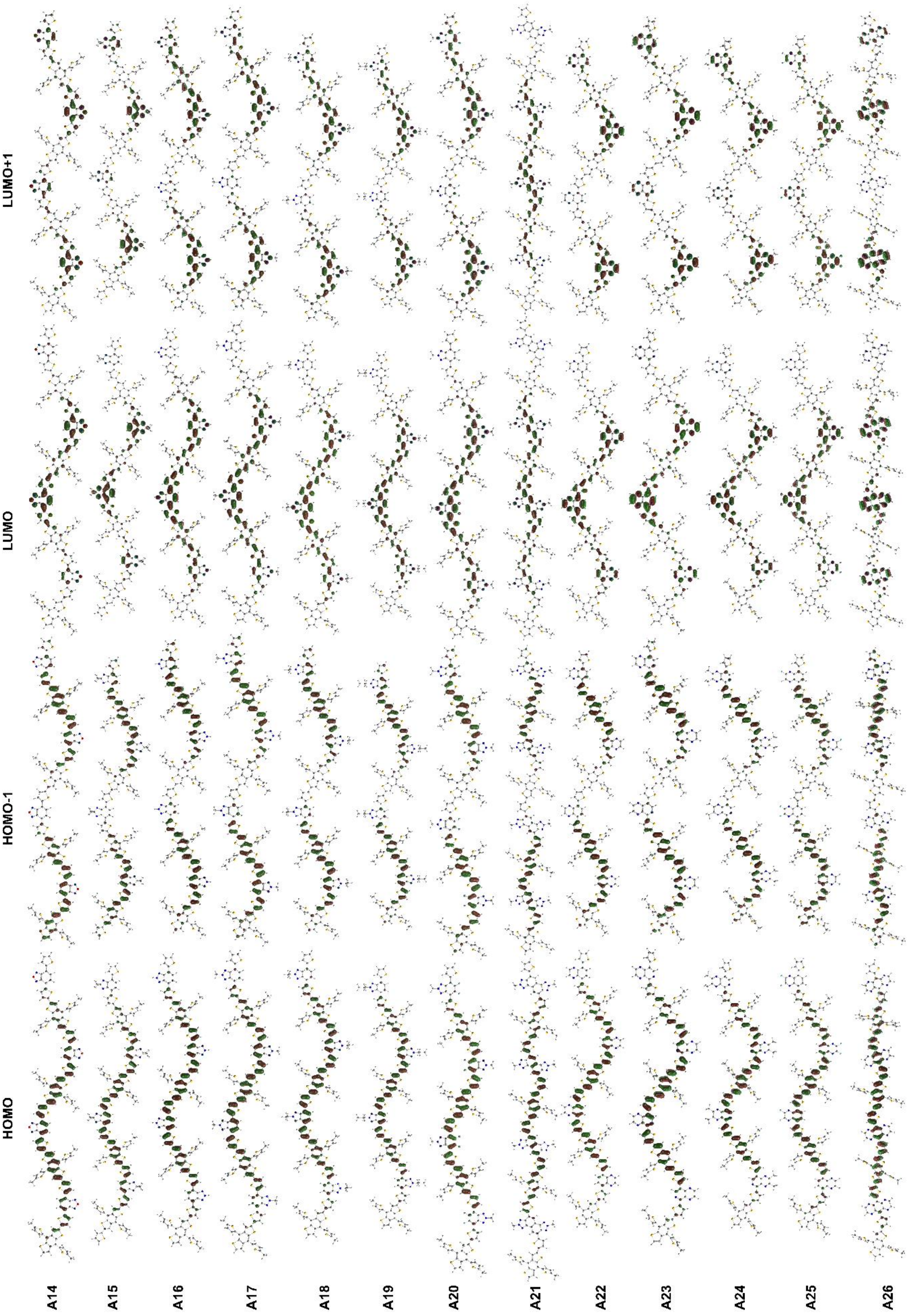

Continue Figure S2

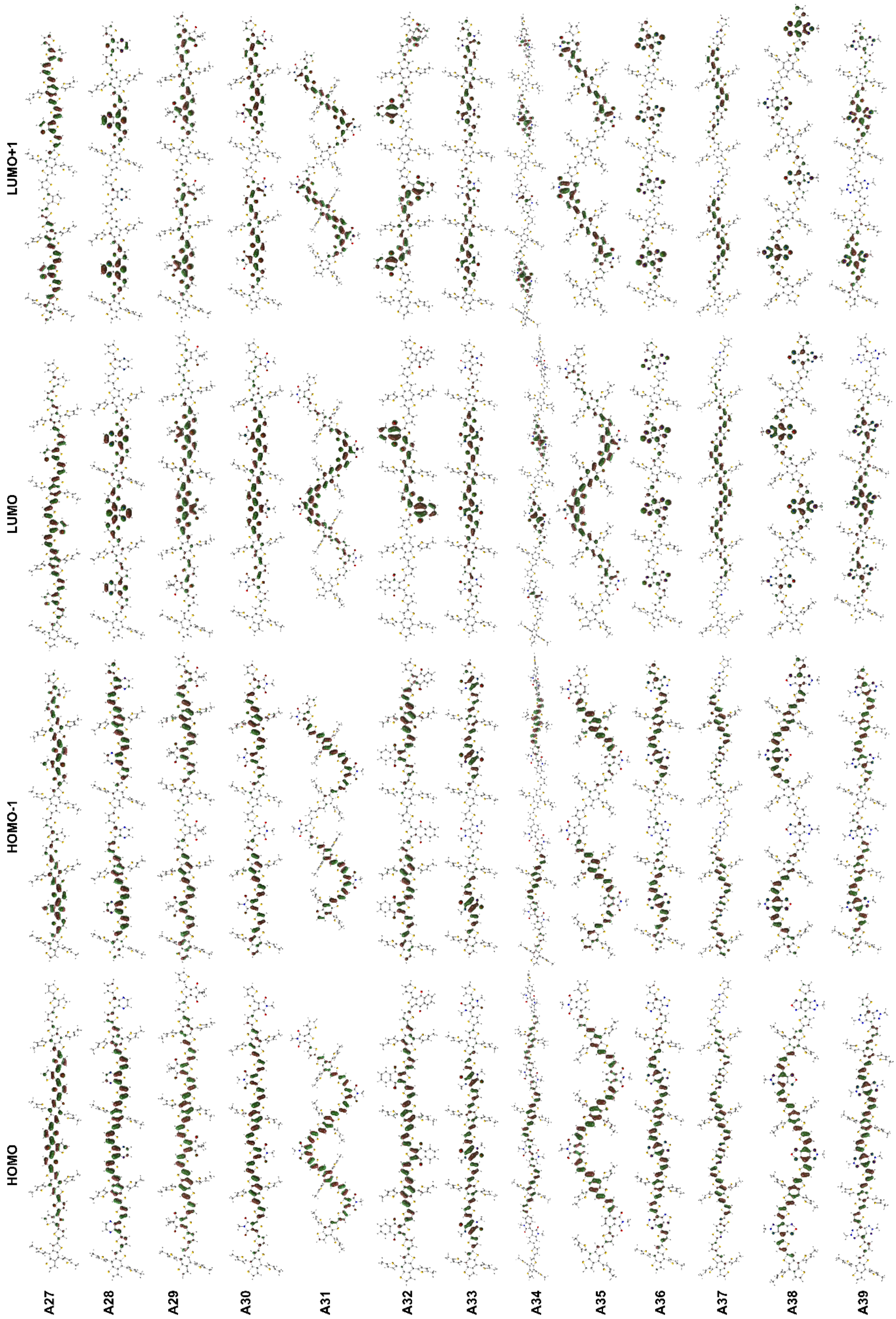

Continue Figure S2

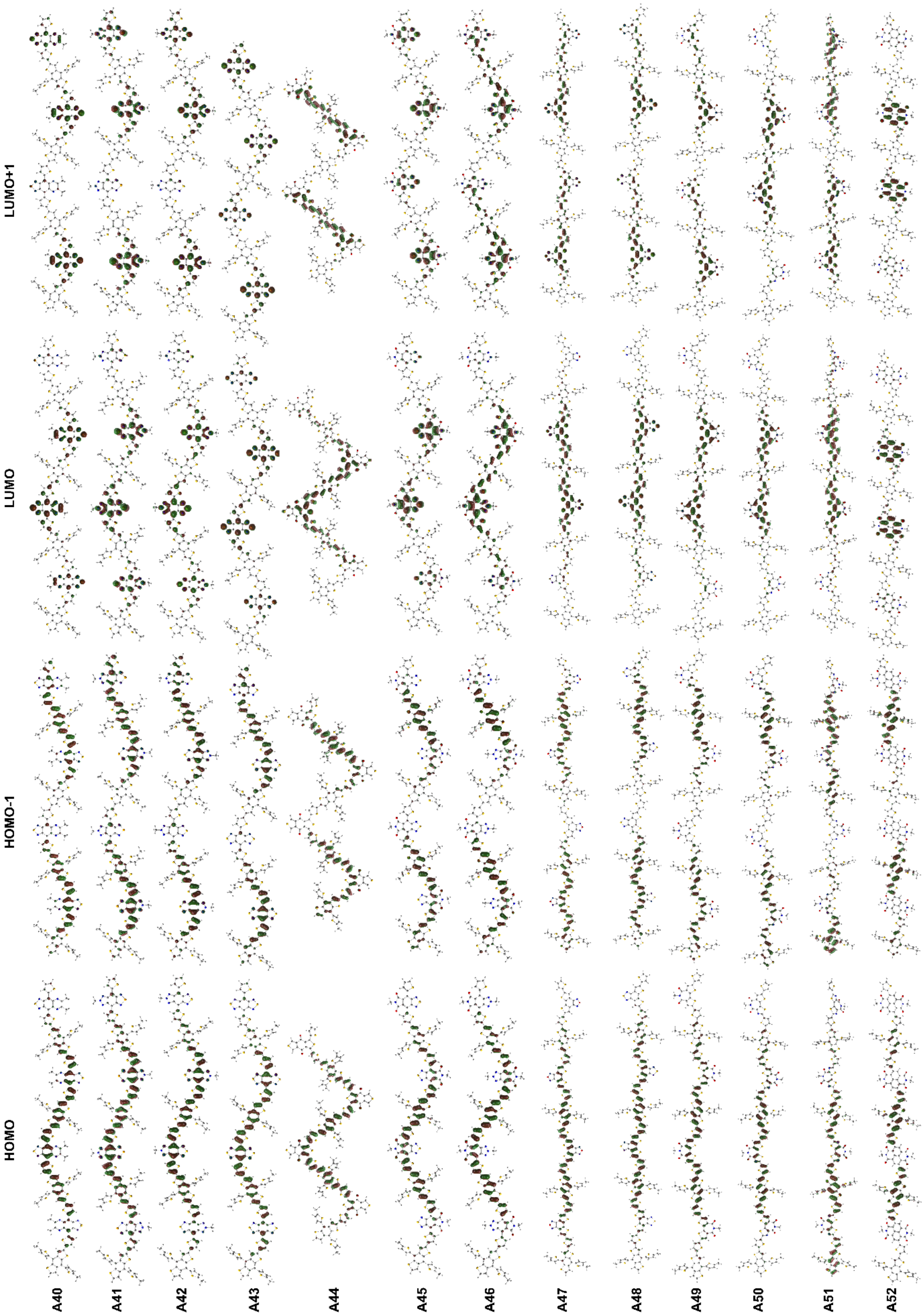

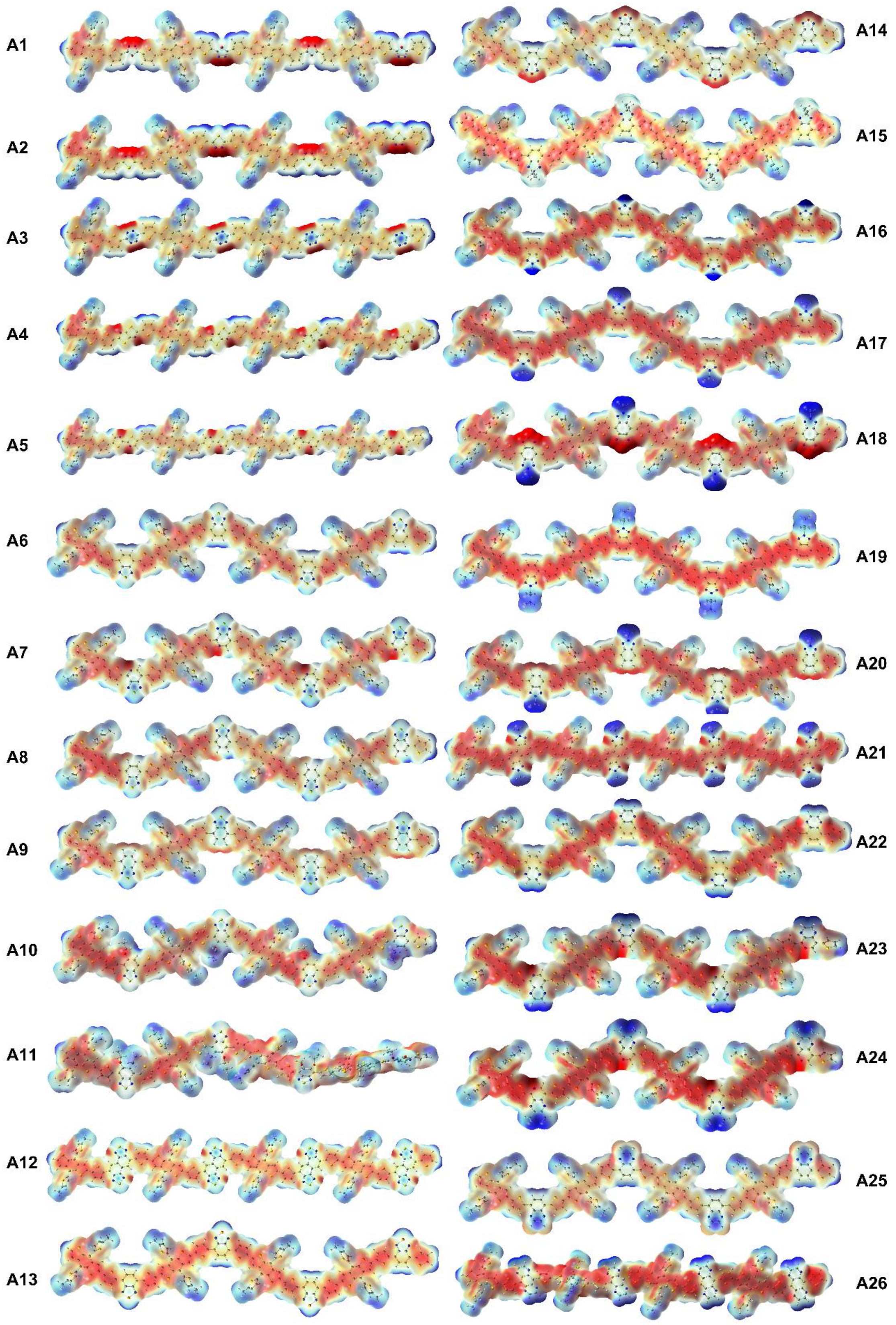

Figure S3 ESP of the tetramers of 52 acceptor units.

Continue Figure S3

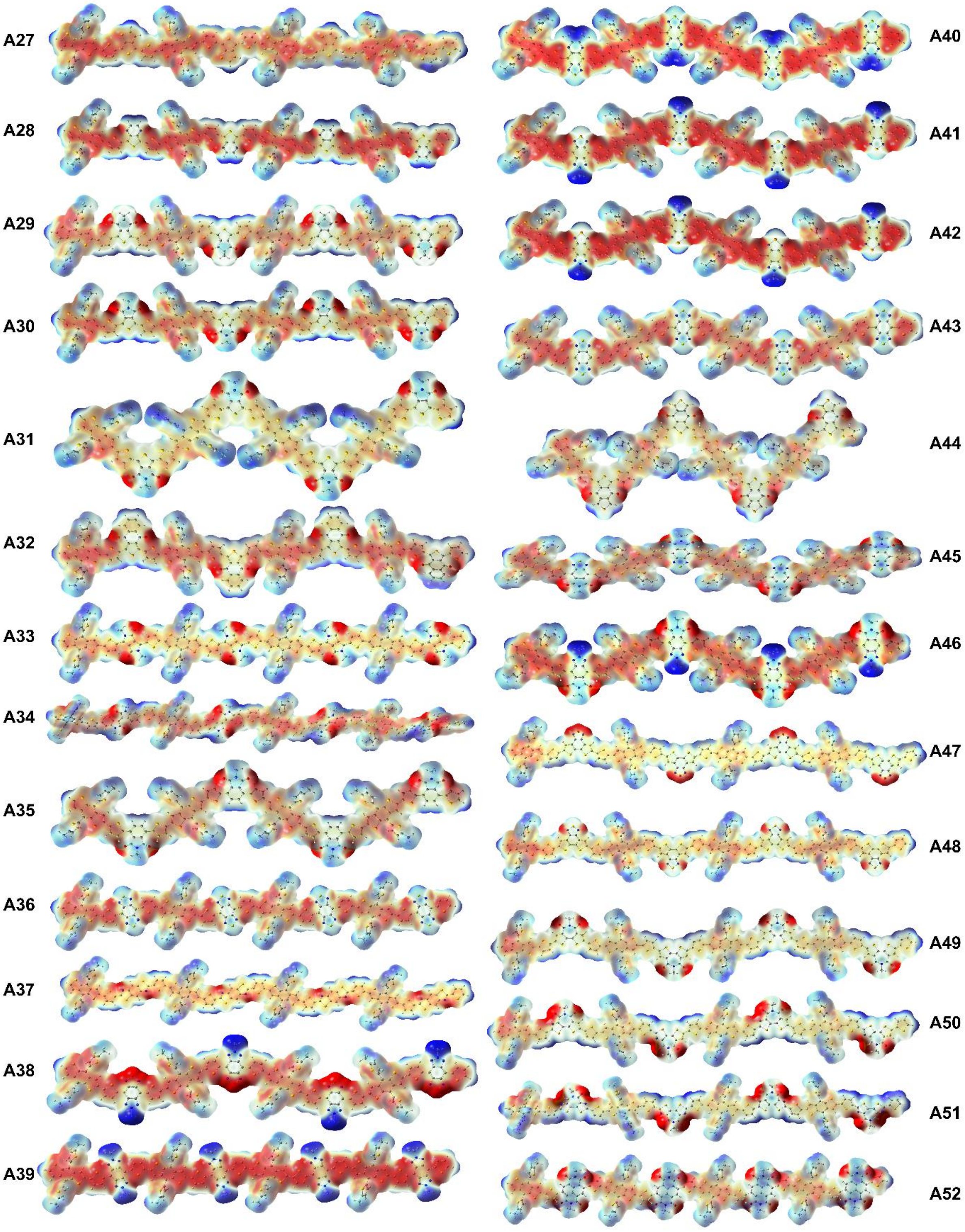

Table S1 Dihedral angles between the acceptor, donor and bridge units. HOMO-LUMO energy levels, bandgap and optical bandgap of the acceptors.

Acceptors	D-B (θ)	A-B (θ)	HOMO (eV)	LUMO (eV)	Bandgap (eV)	Optical Bandgap (eV)
**A1**	17.085	0.620	-5.349	-2.755	2.594	2.243
**A2**	16.625	0.878	-5.285	-2.838	2.446	2.092
**A3**	14.943	0.479	-5.358	-3.055	2.303	1.976
**A4**	15.542	0.918	-5.150	-2.878	2.273	1.933
**A5**	17.132	18.282	-5.277	-2.819	2.459	2.113
**A6**	13.465	6.254	-5.035	-3.082	1.953	1.640
**A7**	13.424	1.920	-5.098	-3.301	1.796	1.498
**A8**	14.680	2.317	-5.065	-3.140	1.924	1.624
**A9**	15.388	0.807	-5.112	-3.161	1.951	1.642
**A10**	14.036	7.237	-4.972	-2.985	1.987	1.672
**A11**	14.730	12.763	-4.939	-2.880	2.059	1.731
**A12**	14.511	6.299	-5.113	-3.274	1.839	1.541
**A13**	13.752	11.114	-4.999	-3.125	1.874	1.560
**A14**	13.426	1.964	-5.169	-3.229	1.940	1.636
**A15**	11.442	1.659	-4.963	-3.213	1.750	1.454
**A16**	14.336	2.289	-4.936	-2.738	2.198	1.871
**A17**	13.884	3.630	-4.883	-2.671	2.212	1.883
**A18**	13.829	2.070	-4.872	-2.659	2.214	1.885
**A19**	13.993	2.040	-4.865	-2.650	2.216	1.887
**A20**	15.284	0.892	-4.974	-2.745	2.229	1.903
**A21**	14.249	4.012	-4.890	-2.655	2.235	1.904
**A22**	16.402	23.332	-5.011	-2.780	2.231	1.888
**A23**	13.324	9.659	-5.031	-3.027	2.004	1.680
**A24**	13.454	11.882	-4.953	-2.820	2.133	1.800
**A25**	16.389	22.369	-5.163	-2.955	2.208	1.870
**A26**	12.020	39.093	-4.822	-3.392	1.430	1.167
**A27**	14.728	19.823	-4.819	-2.681	2.137	1.817
**A28**	12.661	0.706	-4.790	-3.131	1.659	1.377
**A29**	14.860	2.101	-5.145	-3.024	2.121	1.786
**A30**	15.178	1.954	-5.157	-2.878	2.278	1.928
**A31**	11.928	0.949	-5.210	-2.992	2.217	1.874
**A32**	17.507	20.822	-5.126	-2.884	2.242	1.907
**A33**	10.841	0.683	-4.911	-3.179	1.733	1.500
**A34**	13.841	24.026	-5.112	-3.125	1.987	1.699
**A35**	16.909	29.936	-5.215	-2.676	2.539	2.182
**A36**	5.390	0.416	-4.783	-3.853	0.930	0.769
**A37**	16.603	0.737	-5.277	-2.819	2.459	2.108
**A38**	8.223	0.608	-4.802	-3.944	0.858	0.705
**A39**	9.285	0.489	-4.585	-2.984	1.601	1.375
**A40**	10.847	0.086	-4.779	-3.483	1.296	1.062
**A41**	8.377	0.422	-4.671	-3.415	1.256	1.055
**A42**	6.418	0.506	-4.600	-3.498	1.102	0.915
**A43**	4.907	0.329	-4.786	-3.854	0.931	0.770
**A44**	20.568	42.631	-5.299	-3.601	1.698	1.384
**A45**	16.176	31.651	-5.172	-3.308	1.865	1.538
**A46**	15.061	30.768	-5.104	-2.841	2.263	1.913
**A47**	18.019	19.473	-5.122	-2.814	2.308	1.965
**A48**	17.682	19.113	-5.126	-2.815	2.311	1.966
**A49**	17.299	15.888	-5.222	-2.999	2.223	1.888
**A50**	16.259	9.602	-5.310	-3.066	2.244	1.908
**A51**	16.386	1.169	-5.390	-3.136	2.254	1.924
**A52**	20.170	89.696	-5.372	-3.714	1.658	1.384

Table S2 ESP and NBO charges of units in tetramers.

	ESP			NBO		
Acceptors	Acceptor	Bridge	Donor	Acceptor	Bridge	Donor
**A1**	0.322	-0.084	-0.186	-0.108	0.047	0.014
**A2**	-0.112	0.055	0.002	-0.114	0.051	0.012
**A3**	-0.114	0.048	0.018	-0.204	0.090	0.024
**A4**	-0.119	0.062	-0.006	-0.091	0.043	0.004
**A5**	-0.004	0.055	-0.084	-0.040	0.025	-0.009
**A6**	0.087	0.002	-0.088	-0.124	0.067	-0.012
**A7**	0.114	0.007	-0.071	-0.174	0.067	0.004
**A8**	-0.033	-0.012	0.007	-0.153	0.075	-0.004
**A9**	-0.139	0.080	-0.008	-0.173	0.086	0.001
**A10**	-0.056	0.006	0.006	-0.117	0.066	-0.023
**A11**	-0.158	0.111	-0.117	-0.110	0.070	-0.240
**A12**	-0.052	0.057	-0.055	-0.154	0.077	0.000
**A13**	-0.405	0.002	-0.030	-0.118	0.066	-0.015
**A14**	-0.558	0.054	0.051	-0.152	0.074	0.002
**A15**	0.049	0.016	-0.067	-0.147	0.077	-0.009
**A16**	0.059	-0.011	-0.038	-0.092	0.057	-0.023
**A17**	0.082	0.012	-0.071	-0.081	0.054	-0.027
**A18**	0.078	-0.004	-0.051	-0.079	0.053	-0.028
**A19**	-0.331	-0.016	-0.052	-0.078	0.053	-0.029
**A20**	-0.193	0.084	0.019	-0.420	0.073	-0.014
**A21**	0.034	0.033	-0.088	-0.091	0.059	-0.027
**A22**	-0.045	0.067	-0.090	-0.101	0.062	-0.023
**A23**	-0.022	-0.002	-0.022	-0.148	0.061	-0.008
**A24**	-0.003	-0.003	-0.034	-0.121	0.054	-0.018
**A25**	-0.034	0.062	-0.074	-0.122	0.066	-0.011
**A26**	-0.158	0.088	-0.016	-0.198	0.107	-0.014
**A27**	-0.017	0.032	-0.036	0.005	0.011	-0.031
**A28**	0.540	0.054	-0.022	0.648	0.041	-0.023
**A29**	-0.114	0.105	-0.076	-0.165	0.075	0.015
**A30**	-0.187	0.086	0.014	-0.154	0.070	0.013
**A31**	-0.182	0.081	0.016	-0.155	0.072	0.010
**A32**	-0.122	0.080	-0.019	-0.173	0.083	0.007
**A33**	-0.104	0.107	-0.099	-0.183	0.086	0.011
**A34**	0.156	-0.041	-0.009	-0.070	0.043	-0.016
**A35**	0.064	-0.004	-0.057	-0.144	0.075	-0.008
**A36**	-0.207	0.138	-0.074	-0.272	0.028	0.017
**A37**	-0.036	0.073	-0.114	-0.118	0.053	0.011
**A38**	-0.328	0.1338	0.0374	-0.293	0.128	0.0367
**A39**	-0.134	0.125	-0.098	-0.123	0.081	-0.038
**A40**	-0.402	0.081	-0.026	-0.412	0.102	-0.018
**A41**	-0.085	0.053	-0.044	-0.190	0.101	-0.012
**A42**	-0.083	0.050	-0.017	-0.189	0.102	-0.015
**A43**	-0.165	0.103	-0.029	-0.272	0.127	0.017
**A44**	-0.187	0.090	0.013	-0.164	0.079	0.01
**A45**	-0.191	0.085	-0.087	-0.037	0.122	0.008
**A46**	0.351	0.065	-0.025	0.133	0.108	-0.011
**A47**	-0.062	0.035	-0.009	-0.053	0.651	-0.011
**A48**	-0.085	0.061	-0.026	-0.037	0.024	-0.011
**A49**	-0.069	0.037	0.004	0.123	0.020	0.000
**A50**	-0.062	0.064	-0.024	-0.109	0.035	0.014
**A51**	0.004	0.036	-0.082	-0.154	0.063	0.027
**A52**	0.126	-0.042	-0.077	-0.199	0.093	0.010

Table S3 ESP and NBO charges of acceptors. structures were designed as monomer and tetramer. NBO and ESP charges were assigned to the units.

	tetramer-ESP			Tetramer-NBO			monomer-ESP			monomer-NBO		
	A	B	D	A	B	D	A	B	D	A	B	D
1	0.322	-0.084	-0.186	-0.108	0.047	0.014	0.297	-0.221	-0.077	-0.108	0.096	0.012
6	0.087	0.002	-0.088	-0.124	0.067	-0.012	-0.032	0.074	-0.041	-0.121	0.125	-0.004
7	0.114	0.007	-0.071	-0.174	0.067	0.004	0.023	0.027	-0.050	-0.170	0.160	0.010
15	0.049	0.016	-0.067	-0.147	0.077	-0.009	0.000	0.060	-0.060	-0.132	0.136	-0.004
16	0.059	-0.011	-0.038	-0.092	0.057	-0.023	-0.004	0.048	-0.045	-0.092	0.104	-0.012
17	0.082	0.012	-0.071	-0.081	0.054	-0.027	0.023	0.034	-0.058	-0.082	0.097	-0.015
18	0.078	-0.004	-0.051	-0.079	0.053	-0.028	0.046	0.023	-0.069	-0.080	0.095	-0.015
21	0.034	0.033	-0.088	-0.091	0.059	-0.027	0.027	0.007	-0.034	-0.092	0.107	-0.015
28	0.540	0.054	-0.022	0.648	0.041	-0.023	-0.218	0.276	-0.058	-0.059	0.071	-0.012
34	0.156	-0.041	-0.009	-0.070	0.043	-0.016	0.120	-0.099	-0.021	-0.070	0.076	-0.006
35	0.064	-0.004	-0.057	-0.144	0.075	-0.008	0.117	-0.095	-0.023	-0.142	0.143	-0.002
46	0.351	0.065	-0.025	0.133	0.108	-0.011	-0.130	0.160	-0.030	-0.200	0.204	-0.005
51	0.004	0.036	-0.082	-0.154	0.063	0.027	0.016	0.038	-0.053	-0.151	0.131	0.020
52	0.126	-0.042	-0.077	-0.199	0.093	0.010	0.094	-0.119	-0.024	-0.182	0.171	0.010

Table S4 The distribution percentage of LUMO on acceptor and HOMO on donor units by for Mulliken, Stout-Politzer and Ros-Schuit method.

	LUMO-Acceptor (%)			HOMO-Donor (%)		
**Acceptors**	**Mulliken**	**Stout-Polizer**	**Ros-Schuit**	**Mulliken**	**Stout-Polizer**	**Ros-Schuit**
**A1**	64.09	65.06	65.48	87.23	85.99	86.53
**A2**	38.38	39.64	38.63	85.42	84.40	84.73
**A3**	56.75	57.45	55.61	71.51	72.67	71.49
**A4**	71.38	70.02	74.80	75.81	75.62	74.30
**A5**	43.66	44.55	42.78	76.42	75.45	76.88
**A6**	47.24	47.71	47.29	77.48	76.85	77.15
**A7**	53.92	55.75	53.92	84.51	83.42	84.64
**A8**	67.32	68.06	67.15	83.11	82.16	83.35
**A9**	60.33	61.31	61.19	83.75	82.62	83.87
**A10**	73.40	72.63	72.49	88.05	86.77	86.66
**A11**	53.61	54.66	55.09	84.58	83.40	83.44
**A12**	57.39	58.09	57.34	75.44	75.00	74.58
**A13**	54.86	55.75	55.67	79.90	78.85	78.84
**A14**	53.84	54.52	55.72	72.81	73.10	71.69
**A15**	37.40	38.81	36.20	54.83	55.68	55.04
**A16**	34.52	35.54	34.24	81.38	80.56	81.72
**A17**	62.49	63.46	63.68	85.63	84.42	85.26
**A18**	52.55	52.55	52.20	74.27	73.85	73.40
**A19**	5.61	5.94	5.47	97.18	96.64	97.17
**A20**	27.50	30.62	31.41	91.03	89.59	86.91
**A21**	34.99	36.00	34.46	81.55	80.72	81.95
**A22**	76.16	76.40	73.10	82.24	81.63	78.64
**A23**	16.13	15.40	14.09	90.35	89.30	90.09
**A24**	62.53	62.17	64.16	74.34	74.54	73.05
**A25**	30.29	30.77	29.69	90.44	88.88	89.70
**A26**	34.36	35.37	34.13	81.29	80.48	81.64
**A27**	66.07	66.68	67.67	86.64	85.37	86.42
**A28**	54.22	54.93	52.30	86.50	85.10	85.94
**A29**	70.07	69.62	72.43	73.49	73.74	72.43
**A30**	67.25	66.90	69.96	75.10	75.06	73.81
**A31**	26.95	27.62	27.54	90.20	89.08	89.86
**A32**	31.19	32.62	30.21	94.74	93.42	94.35
**A33**	31.48	32.20	31.81	78.49	77.30	76.92
**A34**	60.03	61.40	61.57	76.00	76.88	74.93
**A35**	30.53	31.63	31.95	81.78	80.86	81.72
**A36**	32.82	34.08	34.39	83.26	82.42	82.99
**A37**	71.73	71.20	73.52	78.90	78.16	77.94
**A38**	36.37	37.49	35.82	82.21	81.29	82.65
**A39**	27.58	27.89	28.06	70.16	71.81	67.26
**A40**	48.95	49.43	49.44	86.75	85.37	85.50
**A41**	61.92	62.78	60.81	88.38	86.99	88.21
**A42**	55.20	56.17	54.18	87.90	86.64	87.73
**A43**	72.53	71.81	71.73	82.54	81.50	81.29
**A44**	61.78	62.51	66.14	84.13	83.15	82.01
**A45**	60.71	61.65	62.19	83.71	82.76	80.28
**A46**	71.33	69.98	74.77	75.81	75.62	74.27
**A47**	85.27	85.54	82.79	92.95	91.71	91.13
**A48**	41.68	43.23	43.74	83.42	82.30	80.81
**A49**	45.01	46.37	52.89	57.09	55.54	49.35
**A50**	31.50	31.85	30.20	81.67	80.30	81.81
**A51**	54.00	54.38	53.89	71.72	71.53	71.09
**A52**	74.29	74.67	75.28	81.49	80.17	81.56

Table S5 Dipole moment, polarizability, anisotropic polarizability, hyperpolarizability of the acceptor in tetramers.

Acceptors	dipole moment (Debye)	polarizability (a.u)	Anisotropic Polarizability (a.u)	hyperpolarizability
**A1**	4.19	565.79	558.01	4111.89
**A2**	3.32	600.50	632.57	9122.05
**A3**	1.04	613.05	673.82	17446.06
**A4**	0.50	691.95	819.24	6868.05
**A5**	0.29	731.30	894.33	11031.12
**A6**	0.31	687.53	770.24	16467.14
**A7**	0.98	700.65	806.45	31821.51
**A8**	1.45	690.43	776.62	24836.97
**A9**	1.58	689.26	772.91	21859.69
**A10**	2.02	712.30	758.23	8546.95
**A11**	1.54	728.17	712.92	8410.03
**A12**	0.36	807.92	929.51	31835.27
**A13**	0.34	696.67	768.76	18193.64
**A14**	2.81	679.47	792.99	20621.96
**A15**	0.14	729.27	815.94	23903.21
**A16**	1.62	666.04	745.86	4343.60
**A17**	2.52	681.04	742.23	2750.52
**A18**	2.69	694.15	738.34	2461.87
**A19**	2.83	707.18	736.37	2136.07
**A20**	4.34	681.15	740.98	8212.79
**A21**	0.64	918.52	1092.31	7871.95
**A22**	1.27	665.86	690.24	7948.02
**A23**	2.79	675.82	707.66	16548.52
**A24**	4.15	698.17	694.03	11036.78
**A25**	2.03	666.94	691.37	11757.13
**A26**	0.41	788.97	929.78	40560.83
**A27**	1.51	679.08	735.41	3908.17
**A28**	0.60	720.65	849.13	12830.25
**A29**	3.23	705.44	734.51	13157.13
**A30**	2.68	680.71	721.26	9404.77
**A31**	2.00	683.23	700.97	9670.08
**A32**	1.49	741.08	753.48	13160.56
**A33**	0.77	752.52	916.13	7835.44
**A34**	0.89	937.60	1255.51	44614.46
**A35**	1.53	659.02	648.71	8295.75
**A36**	0.90	870.81	1198.96	74489.53
**A37**	0.63	751.61	912.21	9045.01
**A38**	6.55	874.48	1230.79	75262.66
**A39**	0.90	787.39	946.51	10146.32
**A40**	1.92	830.42	990.75	43448.11
**A41**	3.46	818.62	1041.77	37908.49
**A42**	2.83	844.63	1089.60	43662.18
**A43**	0.92	869.37	1192.59	74506.60
**A44**	1.00	722.69	682.76	14897.79
**A45**	1.10	729.74	758.90	28225.05
**A46**	3.25	714.20	710.93	11745.71
**A47**	4.95	781.36	910.41	10252.11
**A48**	2.44	799.62	905.92	4839.82
**A49**	4.53	803.17	927.04	17555.07
**A50**	5.21	792.86	915.67	16836.34
**A51**	6.00	783.90	908.73	24611.81
**A52**	0.85	814.75	860.58	62658.59

Table S6 Vertical ionization potential, adiabatic ionization potential, vertical electron affinity, reorganisation energy.

Acceptors	Vertical Ionization Potential (VIP)	Adiabatic Ionization Potential (AIP)	Vertical Electron Affinity (VEA)	Reorganisation Energy eV
**A1**	5.808	-5.764	2.254	0.086
**A2**	5.743	-5.699	2.346	0.087
**A3**	5.811	-5.768	2.563	0.084
**A4**	5.589	-5.547	2.417	0.085
**A5**	5.613	-5.570	2.395	0.084
**A6**	5.495	-5.448	2.603	0.091
**A7**	5.560	-5.515	2.818	0.089
**A8**	5.521	-5.476	2.660	0.090
**A9**	5.568	-5.523	2.681	0.089
**A10**	5.428	-5.378	2.509	0.097
**A11**	5.395	-5.341	2.412	0.099
**A12**	5.537	-5.498	2.836	0.077
**A13**	5.459	-5.410	2.645	0.096
**A14**	5.627	-5.581	2.743	0.090
**A15**	5.420	-5.374	2.746	0.091
**A16**	5.401	-5.356	2.262	0.090
**A17**	5.347	-5.302	2.201	0.088
**A18**	5.336	-5.291	2.191	0.088
**A19**	5.328	-5.283	2.184	0.089
**A20**	5.435	-5.390	2.276	0.090
**A21**	5.320	-5.281	2.223	0.078
**A22**	5.476	-5.430	2.304	0.090
**A23**	5.499	-5.454	2.546	0.089
**A24**	5.419	-5.373	2.355	0.090
**A25**	5.621	-5.577	2.474	0.089
**A26**	5.270	-5.211	2.935	0.111
**A27**	5.276	-5.224	2.208	0.104
**A28**	5.249	-5.198	2.658	0.100
**A29**	5.595	-5.551	2.558	0.087
**A30**	5.608	-5.565	2.407	0.085
**A31**	5.680	-5.637	2.498	0.087
**A32**	5.572	-5.529	2.428	0.085
**A33**	5.359	-5.314	2.721	0.088
**A34**	5.496	-5.456	2.724	0.071
**A35**	5.672	-5.630	2.205	0.083
**A36**	5.206	-5.131	3.418	0.165
**A37**	5.685	-5.649	2.385	0.072
**A38**	5.199	-5.145	3.103	0.112
**A39**	5.034	-4.989	2.532	0.088
**A40**	5.225	-5.172	3.031	0.102
**A41**	5.119	-5.070	2.961	0.100
**A42**	5.041	-4.987	3.050	0.113
**A43**	5.213	-5.140	3.416	0.161
**A44**	5.701	-5.662	2.409	0.078
**A45**	5.622	-5.575	2.834	0.092
**A46**	5.559	-5.513	2.376	0.090
**A47**	5.639	-5.597	2.503	0.084
**A48**	5.533	-5.489	2.394	0.087
**A49**	5.628	-5.587	2.571	0.082
**A50**	5.715	-5.676	2.639	0.078
**A51**	5.791	-5.745	2.706	0.078
**A52**	5.717	-5.673	3.164	0.087
